# Hierarchy of the Constituent Characters in Chinese Non-Idiomatic Three-Character Words: An Eye-Tracking Study

**DOI:** 10.3390/bs16091602

**Published:** 2026-09-09

**Authors:** Jinyan Lv, Degao Li, Shuangshuang Wang

**Affiliations:** 1 College of Chinese Language and Literature, Qufu Normal University, Qufu 273165, China; jinyan_lv90@163.com (J.L.); li-degao@163.com (D.L.); 2 Faculty of Psychology, Tianjin Normal University, Tianjin 300387, China

**Keywords:** Chinese three-character words, hierarchical structure, eye tracking, familiarity, predictability

## Abstract

The internal structure of Chinese three-character words may guide eye movements, but whether character-level eye movements vary with hierarchical roles remains unclear. In an eye-tracking lexical decision task, we manipulated branching direction and whole-word familiarity in non-idiomatic three-character words (H3CWs), controlled final-character predictability in the primary analyses, and measured skipping probability and fixation durations at the initial and final positions. Initial characters were skipped less often in right-branching than left-branching H3CWs, whereas final characters showed the reverse pattern, consistent with first-level characters being skipped less often at both positions. The predicted low-familiarity branching difference was reliable only for initial-character total fixation duration; first-fixation and gaze-duration contrasts were inconclusive, and no branching-related duration difference was detected for the final character. Higher predictability increased final-character skipping in left-branching but not right-branching H3CWs and was associated with shorter final-character total fixation durations. Apparent final-character familiarity effects were nonsignificant after predictability was controlled. The findings are compatible with structure-sensitive saccade-target selection and a familiarity-dependent pattern in later fixation time. However, because hierarchical role is intrinsically linked to branching and position, and the proposed mechanisms were inferred rather than measured directly, the pattern cannot be attributed uniquely to hierarchical role or a specific processing mechanism.

## 1. Introduction

Hierarchical organization is a fundamental property of human language and the mental lexicon. Morphological and psycholinguistic accounts propose that complex words are represented not merely as linear strings but as structured combinations of constituents (Bertram et al., 2011; Libben, 2006; Selkirk, 1982; Y. Song et al., 2019). Trimorphemic compounds, as the smallest compounds that permit more than one level of internal grouping, therefore provide a useful test of how constituent structure shapes lexical processing.

Most evidence for hierarchical morphological processing comes from alphabetic languages, in which constituent boundaries may be cued by affixes or hyphens and the linguistic status of those elements remains theoretically debated (Baayen et al., 2019; Blevins, 2016; Sandra, 2021). Chinese provides a complementary case. Because Chinese words are predominantly formed through compounding, hierarchical three-character words (H3CWs) can contain three meaningful constituent characters without overt affixal or spacing cues (Xing, 2006; Yin et al., 2022; Zhou & Marslen-Wilson, 1995). They therefore allow hierarchical roles to be compared across character constituents while reducing asymmetries associated with roots and affixes.

Research on Chinese two-character compounds supports contributions from both character-level and whole-word information, with their relative influence varying according to lexical frequency and task demands (Cui et al., 2021; Hyönä et al., 2024; Sui et al., 2025; Wei et al., 2023). Behavioral and computational evidence further indicates that readers combine constituent meanings when processing familiar as well as novel Chinese compounds (Hsieh et al., 2025). However, these studies have focused mainly on whether compounds are processed through their constituents or as whole words, rather than on how more than two constituents are hierarchically combined.

**Figure 1:**
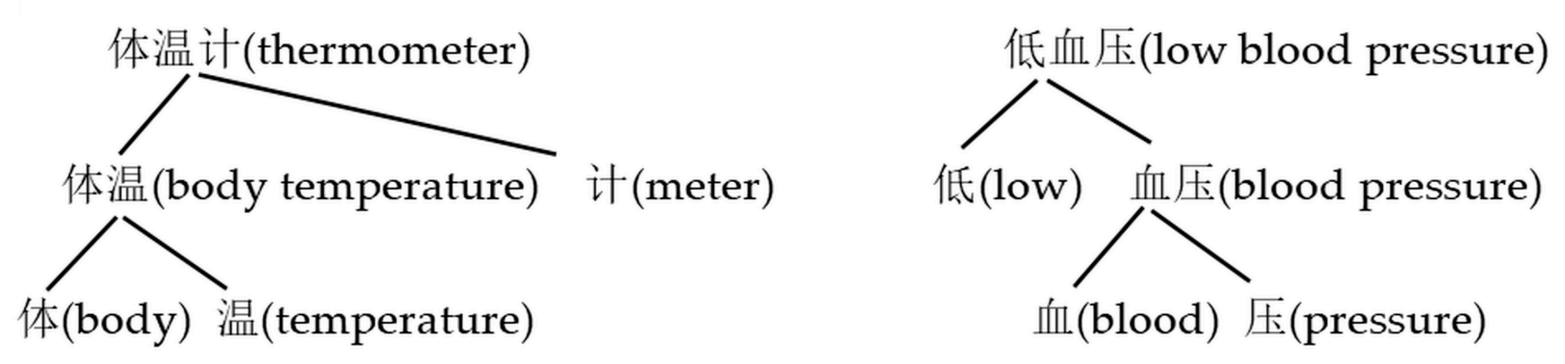
The hierarchical structure of a Chinese LB (**left**) and RB (**right**).

A three-character compound may contain either two morphemes (e.g., 毛玻璃, frosted glass) or three morphemes (e.g., 体温计, thermometer). The present study focuses on the latter type, in which each character retains an independent semantic contribution. Such H3CWs can be represented as left-branching (LB) or right-branching (RB) structures. As illustrated in Figure 1, an LB such as [体温]-计 contains an embedded two-character constituent followed by a first-level character constituent, whereas an RB such as 低-[血压] (low blood pressure) contains a first-level character followed by an embedded two-character constituent. H3CWs thus make it possible to test not only whether constituent information is activated, but also whether character-level processing is sensitive to hierarchical rather than merely linear organization.

### 1.1. Literature Review

Studies of alphabetic trimorphemic words indicate that processing is sensitive to major constituent boundaries. Visual segmentation facilitates recognition when it coincides with a major morphological boundary but disrupts processing when it divides an embedded constituent (Bertram et al., 2011), and morphological priming is stronger for genuine constituents than for linearly overlapping non-constituent substrings (Y. Song et al., 2019). At the same time, interpretations of structurally ambiguous words can be influenced by sentence context (de Almeida & Libben, 2005; Pollatsek et al., 2010). Together, these findings suggest that an embedded bimorphemic constituent can function as an intermediate processing unit, although its contribution is sensitive to lexical and contextual conditions.

Alphabetic and Chinese writing differ substantially in their orthographic organization, but the preceding evidence yields a shared processing prediction: a trimorphemic word may be processed as a larger embedded constituent combined with another constituent, rather than as three equivalent units in a linear sequence. In alphabetic languages, affixes, letter sequences, and hyphens may provide cues to constituent boundaries. Chinese characters, by contrast, are visually discrete units with clear character boundaries. However, the equally spaced characters within an H3CW do not overtly indicate whether the internal structure is [AB]-C or A-[BC]. Chinese H3CWs therefore provide a particularly informative test of whether readers derive hierarchical constituent groupings in the absence of explicit visual markers of word-internal morphological boundaries.

Evidence from East Asian morphographic scripts is broadly compatible with this prediction. Miwa et al. (2017) reported structure-sensitive eye-movement patterns in Japanese trimorphemic compounds, and Yin et al. (2022) found an LB advantage in Chinese lexical decision. More recently, Cui et al. (2026) obtained evidence for both compositional and holistic processing of Chinese three-character compounds: first-character frequency influenced lexical decisions and sentence-reading eye movements, whereas optimally located single fixations showed less evidence of constituent-frequency effects. The contribution of internal structure also differed between isolated lexical decision and sentence reading, indicating that three-character compound processing is sensitive to task and reading context.

Recent Chinese sentence-reading studies further indicate that internal structure can influence eye-movement control. Luo et al. (2023) found that morphological structure affected initial fixation location, while Yan et al. (2026) showed that parafoveal grouping influenced both fixation placement and subsequent target-word processing. Using overlapping ambiguous three-character strings rather than lexicalized compounds, M. Song et al. (2026) additionally observed an AB-C segmentation advantage and found that predictability primarily affected skipping. These findings are consistent with recent models that emphasize dynamically coordinated character- and word-level processing in Chinese eye-movement control (Liu et al., 2025). However, they mainly concern eye movements on the word or string as a whole. It remains unclear whether the hierarchical status of an individual character is associated with the likelihood and duration of direct fixation.

Whole-word familiarity may further modulate these character-level differences. Familiarity influences the recognition of Chinese two-character words, multi-character words, and idiomatic expressions (Fu et al., 2022; Su et al., 2023; Tsang et al., 2018). Dual-route accounts propose that stronger whole-word representations facilitate whole-word access, whereas weaker representations increase the contribution of constituent-level information (Caramazza et al., 1988). Accordingly, branching-related differences between first- and second-level characters may be greater for low-familiarity than for high-familiarity H3CWs. In the present study, this proposal is tested as a modulation of observable eye-movement differences; holistic and decompositional processing remain theoretical interpretations rather than directly measured mechanisms.

### 1.2. The Present Study

To address these gaps, the present study used a lexical decision task combined with eye-tracking to examine how attention allocation to constituent characters in H3CWs is jointly modulated by branching direction and whole-word familiarity. The lexical decision task required participants to process each three-character string sufficiently to determine whether it constituted a meaningful word. Because the H3CWs were presented in isolation, this judgment was not supported by sentence context and could therefore draw on information from both the whole word and its constituent characters. Eye tracking further revealed how visual attention was distributed across character positions: skipping probability indexed whether a character was directly fixated, whereas fixation-duration measures reflected processing after fixation. Together, these measures allowed us to examine where hierarchy-related differences occurred and at what relative stage they became observable. Three hypotheses were thus proposed.

Hypothesis 1 concerned whether character-level eye-movement behavior is sensitive to hierarchical status. If first-level constituents attract more attention than second-level characters embedded within a two-character constituent, the direction of the branching effect should reverse across character positions. At the initial-character position, the initial character is a first-level constituent in RBs but a second-level constituent in LBs; therefore, it should be skipped less often and, when directly fixated, receive longer fixation durations in RBs than in LBs. At the final-character position, the final character is a first-level constituent in LBs but a second-level constituent in RBs; consequently, it should be skipped less often and receive longer fixation durations in LBs than in RBs.

Hypothesis 2 concerned the time course of these hierarchy-related differences. If hierarchical status affects early saccade-target selection and initial character processing, the position-specific branching pattern predicted in Hypothesis 1 should be evident in skipping probability (SP) and/or first-fixation duration (FFD). Effects emerging in gaze duration (GD) or total fixation duration (TFD) would instead indicate that branching-related differences persist into, or become detectable during, later stages of processing. Thus, evidence across the measures was expected to clarify when sensitivity to hierarchical status becomes observable, rather than requiring the effect to appear uniformly in every measure.

Hypothesis 3 concerned whether the predicted branching differences vary with whole-word familiarity. On the basis of dual-route accounts, weaker whole-word representations were expected to increase the contribution of constituent-level information. Accordingly, the position-specific branching differences predicted in Hypothesis 1 were expected to be larger for low-familiarity than for high-familiarity H3CWs. More specifically, for low-familiarity items, the initial character was expected to be skipped less often and/or fixated longer in RBs than in LBs, whereas the final character was expected to show the reverse pattern. For high-familiarity items, these branching differences were expected to be attenuated. This prediction concerns the modulation of observable eye-movement differences; holistic and decompositional processing are treated as possible theoretical explanations rather than as processes directly measured in the present experiment.

## 2. Materials and Methods

### 2.1. Participants

Ninety-three native Chinese-speaking students (65 females, mean age 18.4 years) were recruited on a university campus via flyer advertisement. All participants were native Chinese speakers with normal or corrected-to-normal vision and were unaware of the purpose of the experiment. They provided written informed consent in accordance with the Declaration of Helsinki. This study received ethical approval from the Biomedical Ethics Committee of Qufu Normal University (protocol code 2026092; 23 March 2026). Because no previous study had examined the familiarity × branching interaction in character-level eye movements for Chinese three-character words, no directly applicable empirical effect-size estimate was available. An effect size of *d* = 0.45 was initially considered as an approximate planning value rather than an empirically established estimate. We therefore conducted an approximate sensitivity analysis using PANGEA (Version 0.2; Westfall et al., 2014). Familiarity and branching direction were specified as two-level fixed factors, participants and stimuli as random factors, and stimuli as nested within the four familiarity × branching conditions. Because PANGEA assumes a balanced design, 24 stimuli per condition (96 stimuli in total) were specified as an approximation to the 98 stimuli used in the experiment. With the final analytic sample of 91 participants, one observation per participant–stimulus combination, and PANGEA’s default variance-component assumptions, estimated power was 0.78 for an interaction effect of *d* = 0.28 and 0.81 for an effect of *d* = 0.29. Thus, the final design had approximately 80% power to detect a familiarity × branching interaction of *d* ≈ 0.29 or larger.

### 2.2. Apparatus

Participants’ eye movements were monitored using an SR Research EyeLink 1000 Plus (Ottawa, ON, Canada) with a sampling rate of 2000 Hz. Stimuli were presented in Song font on a single line on a 24-inch Dell CRT monitor with a refresh rate of 144 Hz and a screen resolution of 1024 × 768 pixels. At a viewing distance of 75 cm, each Chinese character corresponded to approximately 1.93 degrees of visual angle.

### 2.3. Materials

All three-character words listed in the *Modern Chinese Dictionary* (7th ed.; Chinese Academy of Social Sciences, Institute of Linguistics, Dictionary Editing Office, 2016) were first selected to establish the initial database. After duplicate entries were removed, we excluded words that (a) contained constituent characters without independent semantic functions (e.g., 毛玻璃, frosted glass), (b) conformed to neither LB nor RB structures (e.g., 马大哈, careless), or (c) whose middle characters could combine with either the initial or final characters to form items that appeared in the SUBTLEX-CH database (e.g., 面包车, minibus; Cai & Brysbaert, 2010). This screening procedure yielded an initial norming database of 2287 H3CWs, including both idiomatic and non-idiomatic items. All 2287 items were included in the subsequent norming tasks.

For each rating task, the H3CWs were divided into 19 lists, and each list was rated by a separate group of approximately 20 participants. Each participant completed only one list, and mutually exclusive groups of participants completed the different rating tasks. Because occasional missing or invalid responses were excluded, the number of valid ratings varied slightly across items; item-level means were therefore calculated from all valid responses available for each item. Familiarity was rated on a 7-point scale (1 = *very unfamiliar*, 7 = *very familiar*), whereas semantic non-literalness was rated on a separate 7-point scale (1 = *very easy to understand through the constituent characters*, 7 = *very difficult to understand through the constituent characters*). The same rating procedure was applied to the embedded two-character constituents. Across the complete norming database, the ratings showed good cross-rater consistency: Cronbach’s α was 0.88 for H3CW familiarity and 0.89 for H3CW non-literalness, and 0.92 for both familiarity and non-literalness ratings of the embedded two-character constituents. For the present experiment, items listed in the *Modern Chinese Proverb and Idiom Standardized Dictionary* (Li, 2011) were classified as idiomatic and excluded. This procedure yielded 1628 non-idiomatic H3CWs, comprising 1372 LBs and 256 RBs. The critical experimental items were subsequently selected from this non-idiomatic subset.

Using the same list assignment described above, the final character of each H3CW was replaced by a blank, and participants supplied the first character that came to mind. Predictability was calculated as the proportion of valid responses that supplied the intended target character. Plausible responses other than the intended target were coded as non-target completions.

Frequency values were log-transformed frequencies from SUBTLEX-CH (Cai & Brysbaert, 2010), and stroke counts were obtained from the *Xinhua Dictionary* (Institute of Linguistics, Chinese Academy of Social Sciences, 2020). Following Yen et al. (2012), a character’s initial and final positional probabilities (IPP and FPP) were calculated as the summed frequency of words containing that character in the initial or final position divided by the summed frequency of all words containing that character. These database measures were then used to select the critical materials for the present experiment while controlling for lexical and sublexical variables.

The resulting 98 critical materials were assigned to four conditions according to their branching and whole-word familiarity: (1) 24 high-familiarity RBs (e.g., 超-[负荷], excess load; *M* = 6.07), (2) 28 high-familiarity LBs (e.g., [外交]-官, diplomatic officer; *M* = 6.24), (3) 23 low-familiarity RBs (e.g., 铁-[合金], iron alloy; *M* = 3.20), and (4) 23 low-familiarity LBs (e.g., [冲击]-波, shock wave; *M* = 2.73). Square brackets indicate the embedded two-character constituent, and the value following each example is its item-level mean familiarity rating.

As indicated in Table 1, high-familiarity H3CWs elicited significantly higher familiarity ratings than low-familiarity ones, *t*_(75)_ = 17.39, *p* < 0.001, 95% CI [1.87, 2.36]. Crucially, within each familiarity level, no significant differences were observed in their respective ratings (*p*s > 0.42). Most lexical and sublexical variables were well-matched across conditions, *F*s < 1.4, *p*s > 0.27. These included the non-literalness of H3CWs; the frequency, familiarity, and non-literalness of the two-character constituents; and the positional probabilities, frequency, and stroke counts of constituent characters. However, the predictability of the final character significantly differed between familiarity conditions, *t*_(96)_ = 8.02, *p* < 0.001, 95% CI [0.29, 0.47]. Consequently, predictability was included as a covariate in subsequent analyses.

To balance the lexical decision task, an equal number of non-word stimuli were generated by replacing the initial, medial, or final characters of one-third of the real three-character compounds in the database that were not selected as critical experimental items. These non-words were checked against the SUBTLEX-CH-WF corpus (Cai & Brysbaert, 2010), and any combinations appearing in the corpus were eliminated. The remaining candidates were also manually inspected by native Chinese speakers to exclude combinations that could be interpreted as established words, common phrases, proper names, or readily acceptable novel compounds.

### 2.4. Procedure

Participants were seated with their chin stabilized to minimize head movement. Calibration was performed using a three-point horizontal procedure, requiring an average validation error of less than 0.20°. A drift check preceded each trial. Crucially, the drift-correction fixation point was positioned 1.93° to the left of the target onset. Given that each character subtended 1.93° of visual angle and that the parafoveal view spans approximately 2° to 5° from the point of fixation, this placement ensured that when the fixation point disappeared, the first two characters of the H3CW fell within the parafoveal region. The target stimulus was then presented at the screen’s center. The stimulus remained on screen for 5000 ms or until the participant responded. Participants were instructed to read the item silently and perform a lexical decision task (judging whether the target was meaningful) by pressing designated keys. Each trial concluded with a 1000 ms blank screen. The experimental session consisted of six practice trials followed by the 98 experimental items and fillers (non-words), which were presented in a randomized order. The entire session lasted approximately 15 min, after which participants received a small monetary reward for participation.

### 2.5. Eye-Movement Measures and Data Analysis

The initial and final characters of each H3CW were defined as separate interest areas. The dependent measures were SP, FFD, GD, and TFD. SP was calculated from all eligible trials, whereas fixation-duration measures were calculated only for trials in which the corresponding interest area was directly fixated. FFD was defined as the duration of the first fixation on an interest area, GD as the sum of all first-pass fixations on that area before leaving it, and TFD as the sum of all fixations on that area, including regressions.

During Data Viewer export, fixations shorter than 80 ms or longer than 1200 ms were excluded using the fixation-duration filters. Trials containing incorrect responses or blinks, as well as trials with response times shorter than 300 ms, were excluded from the eye-movement analyses. Following the trial-level exclusions, observations were standardized separately for each fixation-duration measure within each participant and experimental condition. The original filtering procedure retained observations only when the standardized score was defined and its absolute value was below 3. Initial- and final-character interest areas were analyzed separately. SP was analyzed using logistic generalized linear mixed-effects models with a binomial distribution and logit link, whereas log-transformed FFD, GD, and TFD were analyzed using linear mixed-effects models. Models were fitted using the lme4 package (Bates et al., 2015) in R (Version 4.5.1; R Core Team, 2025). For the fixation-duration models, *p* values were obtained using lmerTest (Version 3.1.3; Kuznetsova et al., 2017) with Satterthwaite’s approximation. Familiarity and branching were entered as fixed effects using successive-difference contrasts, and participants and items were included as crossed random effects. Predictability was entered as a continuous covariate in its original scale. Each model was initially fitted with the maximal random-effects structure justified by the design (Barr et al., 2013) and simplified only when required for convergence or to avoid singularity. The final fixed- and random-effects structures are reported in Table A2; the data, R scripts, and full model-fitting details are available at https://osf.io/6ep2q (accessed on 5 September 2026). Fixation durations were log-transformed before model fitting to reduce positive skew (although comparable results were obtained without this transformation).

Because final-character predictability differed substantially between the familiarity conditions, models including predictability as a covariate were designated as the primary analyses. These models were used to evaluate familiarity and branching effects after accounting for the measured predictability imbalance. Models excluding predictability were treated as unadjusted sensitivity analyses (see Table A3). For statistically significant interactions, the two prespecified follow-up contrasts or simple slopes were treated as a family, and their *p* values were adjusted using the Holm procedure to limit familywise Type I error. The omnibus tests concerned prespecified effects on theoretically distinct eye-movement measures and were not adjusted across measures; accordingly, effects close to the conventional threshold were not treated as substantive evidence. Unless otherwise stated, the results below refer to the primary models controlling for predictability.

## 3. Results

Two participants were excluded from the final analyses: one because of accuracy below 80%, and one because excessive blinking resulted in nearly no valid eye-movement data. The final sample comprised 91 participants. Following participant exclusion, the trial-level criteria described above removed 19.9% of the remaining trials. Condition-specific trial-level exclusions due to incorrect responses, blinks, and response times shorter than 300 ms are reported in Table A1a. The subsequent measure-specific z-score filtering excluded 298 of 15,749 eligible fixation-duration observations (1.89%), with exclusion rates ranging from 1.07% to 3.62% across the six position-by-measure datasets. Condition-specific counts are reported in Table A1b.

Descriptive statistics and the numbers of trial-level observations included in the initial- and final-character models are presented in Table 2. Fewer observations were available for the fixation-duration analyses than for the SP analyses, particularly for the frequently skipped final character.

### 3.1. The Initial Character

#### 3.1.1. Skipping Probability (SP)

In the primary model, initial characters were skipped significantly less often in RBs than in LBs (*b* = −0.13, *SE* = 0.05, *z* = −2.49, *p* = 0.013; Table 3). Neither familiarity, predictability, nor the Familiarity × Branching interaction was significant. The unadjusted sensitivity model yielded the same pattern (Table A3).

#### 3.1.2. Fixation Durations

**Figure 2:**
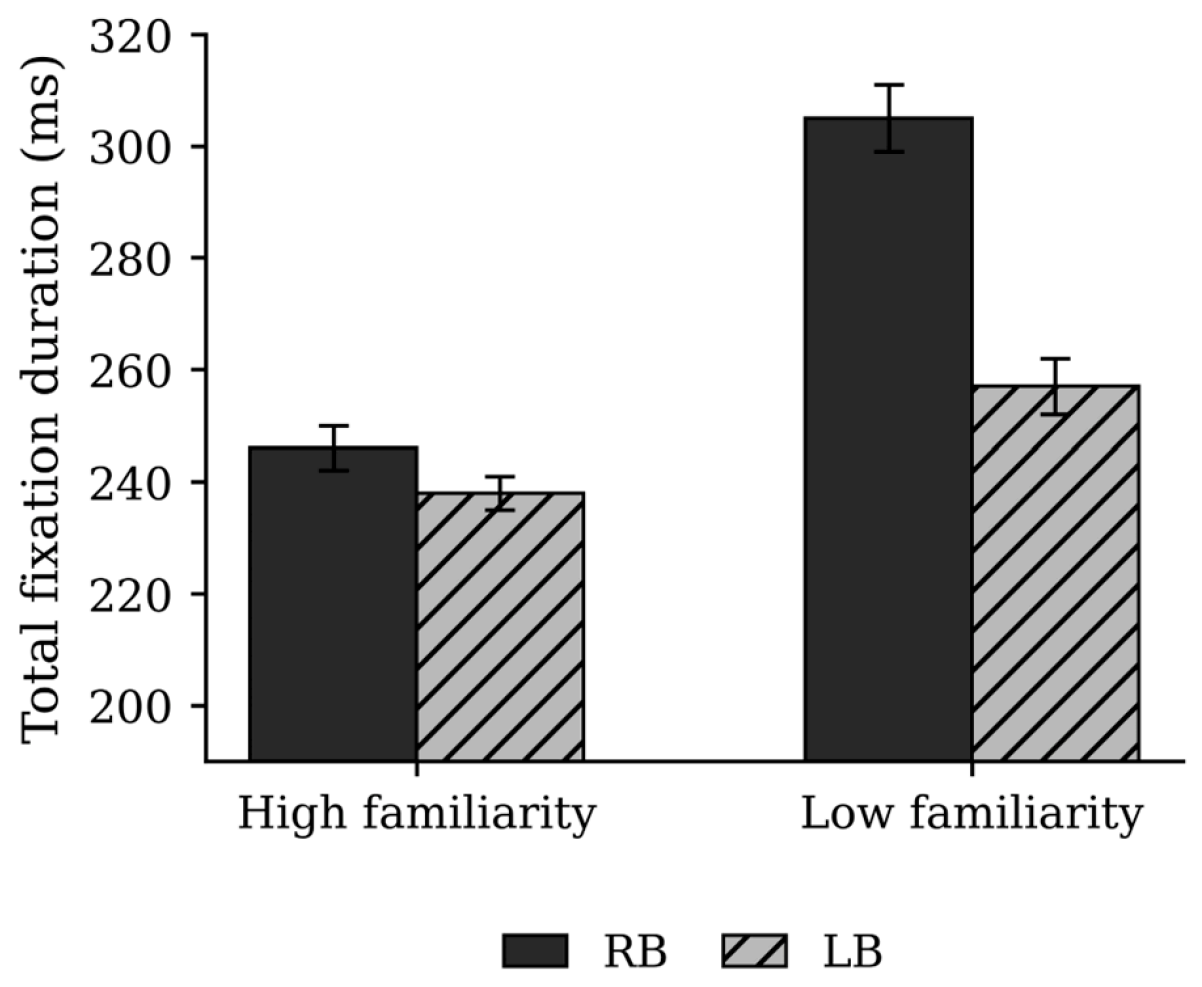
Initial-character TFD as a function of familiarity and branching. Error bars represent standard errors of the mean.

For TFD, the primary model showed significant main effects of familiarity and branching: initial-character fixation times were longer for low- than high-familiarity H3CWs and longer for RBs than for LBs. Significant Familiarity × Branching interactions were also observed in FFD, GD, and TFD (Table 3). Planned contrasts showed that, among low-familiarity H3CWs, initial-character TFD was significantly longer for RBs than LBs (Holm-adjusted *p* < 0.001). The corresponding contrasts did not reach statistical significance in FFD (Holm-adjusted *p* = 0.118) or GD (Holm-adjusted *p* = 0.103) and were treated as inconclusive. Within RBs, initial-character TFD was also significantly longer for low- than high-familiarity H3CWs (Holm-adjusted *p* < 0.001). The TFD interaction is illustrated in Figure 2.

### 3.2. The Final Character

#### 3.2.1. Skipping Probability (SP)

**Figure 3:**
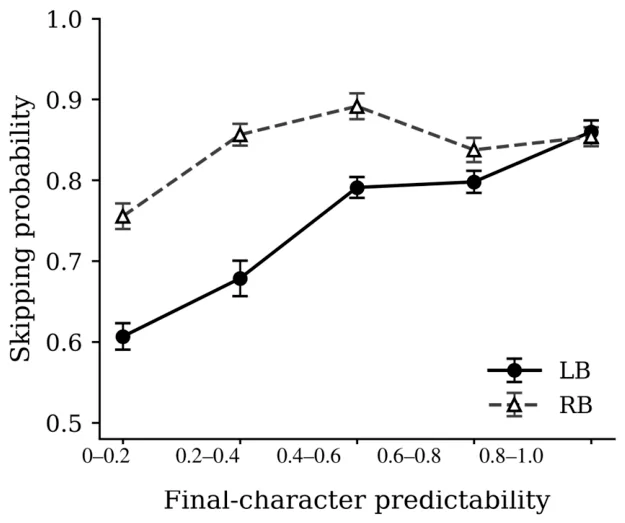
Branching × predictability interaction for final-character SP.

In the primary final-character SP model, final characters were skipped more frequently in RBs than in LBs (*b* = 1.47, *SE* = 0.36, *z* = 4.03, *p* < 0.001; Table 3). This effect was qualified by a significant Branching × Predictability interaction (*b* = −1.58, *SE* = 0.64, *z* = −2.48, *p* = 0.013). Holm-adjusted simple-slope analyses using the emmeans package (Version 2.0.3; Lenth & Piaskowski, 2026) showed that higher predictability increased final-character skipping in LBs (*b* = 1.98, *SE* = 0.55, 95% CI [0.90, 3.06], *z* = 3.60, adjusted *p* < 0.001), whereas the corresponding slope was not significant in RBs (*b* = 0.40, *SE* = 0.48, 95% CI [−0.55, 1.34], *z* = 0.83, adjusted *p* = 0.408). The interaction is illustrated in Figure 3. In the unadjusted sensitivity model, familiarity predicted final-character skipping, but this effect disappeared when predictability was controlled and was therefore not interpreted as an independent familiarity effect (Table A3).

#### 3.2.2. Fixation Durations

For the final-character interest area, none of the familiarity, branching, or interaction terms was significant in FFD or GD (Table 3). In TFD, the familiarity effect observed in the unadjusted sensitivity model was no longer significant after predictability was controlled. The primary model instead showed a negative association between predictability and TFD (*b* = −0.15, *SE* = 0.06, *t* = −2.25, *p* = 0.027), indicating shorter total fixation durations at higher levels of predictability. Because the final-character fixation-duration models were based on only 1463–1465 observations, these null branching effects should be interpreted cautiously.

## 4. Discussion

**Figure 4:**
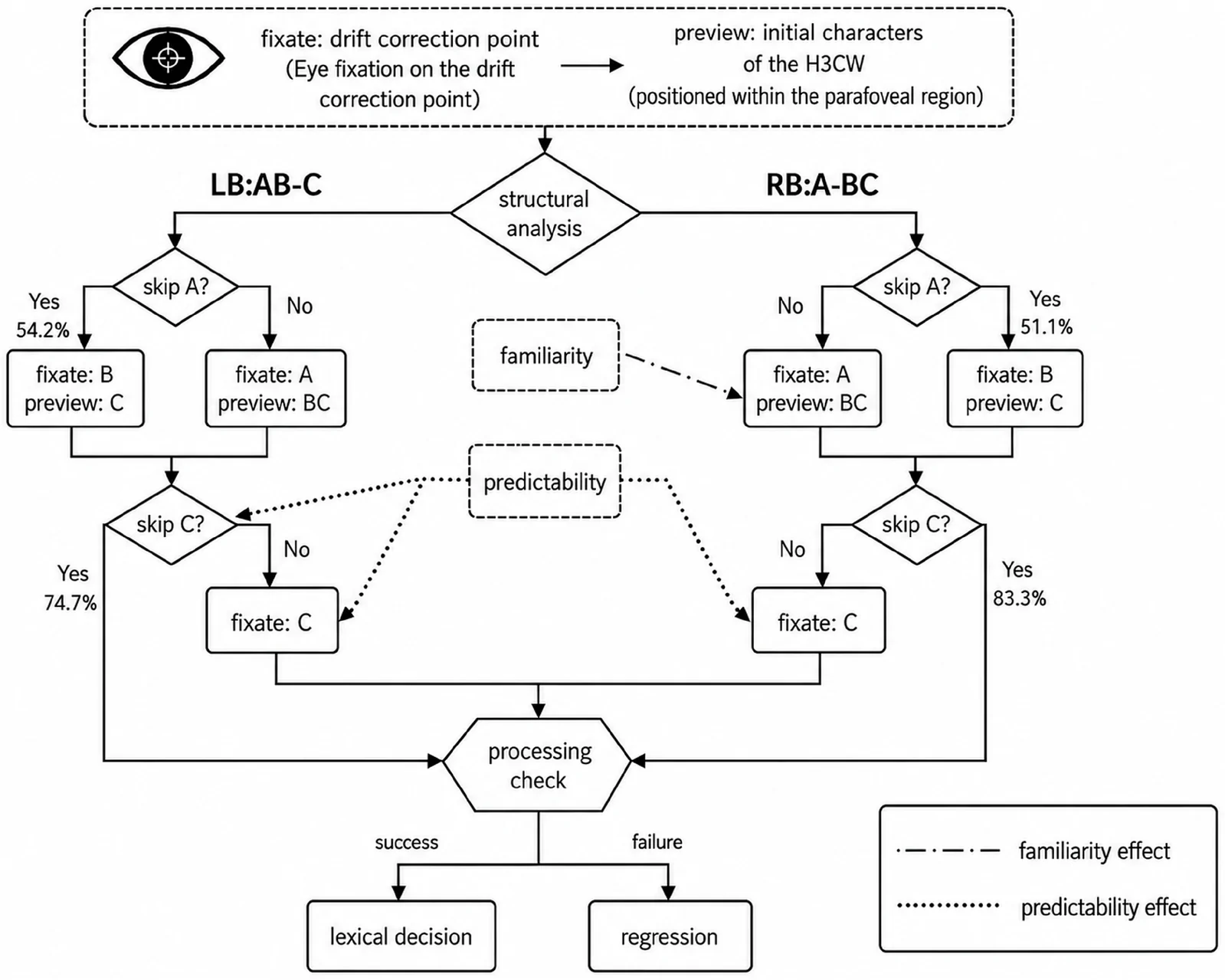
A tentative schematic account of the observed eye-movement patterns in H3CWs. *Note*. Percentages represent observed skipping rates; the arrows and processing sequence are post hoc theoretical interpretations of the eye-movement pattern. Solid arrows depict a possible structure-related processing flow, and dashed arrows depict possible modulation by familiarity and final-character predictability. The diagram summarizes the main first-landing patterns (94.8% of trials with the first landing on either of the first two characters) and omits the relatively infrequent first landings on the final character.

The primary objective of the present study was to investigate whether attention allocation to constituent characters in Chinese H3CWs is organized by hierarchical structure and modulated by lexical familiarity. Participants completed a visual lexical decision task while their eye movements were recorded on the initial- and final-character interest areas. The clearest finding was the reversal of the branching effect in initial- and final-character SP, whereas fixation-duration evidence was more limited. Overall, the findings are compatible with early sensitivity to information associated with branching structure, although the evidence differed across measures. Figure 4 summarizes a tentative explanatory account.

### 4.1. Hierarchical Processing of Constituent Characters

The reversal of the SP effect across character positions is important because it is not consistent with a general, position-invariant advantage for either RBs or LBs. Rather, the character occupying the first-level role was less likely to be skipped at both positions. Because SP reflects whether an interest area receives direct fixation, this pattern suggests that information associated with branching structure was available during saccade-target selection. The fixation-duration results provide a more qualified picture of the time course: evidence for the predicted branching difference was confined to initial-character TFD, whereas the earlier FFD and GD contrasts and all final-character duration measures yielded no reliable corresponding differences. Thus, the data provide clearer evidence for position-specific branching effects in early fixation selection than for their persistence after direct fixation.

This interpretation converges with recent evidence that, during Chinese sentence reading, readers can obtain and use hierarchical structure information in the parafovea to guide saccade programming and facilitate subsequent lexical processing (Luo et al., 2023; Yan et al., 2026). The present findings extend this line of evidence by showing position-specific branching differences in character-level skipping during isolated-word recognition. These differences are compatible with early sensitivity to the structural roles associated with branching. However, because hierarchical role is intrinsically linked to branching direction and character position in these materials, the observed pattern cannot be attributed uniquely to hierarchical role.

A possible explanation comes from flexible saccade-target selection accounts in Chinese reading, according to which readers tend to select the center of a word as the saccade target when parafoveal word segmentation is successful (Yan et al., 2010), and from preferred-viewing-location accounts, which assume that readers tend to target visually or linguistically optimal positions within a processing unit (Li et al., 2011; Ma et al., 2015). On this account, the two second-level characters may be relatively tightly integrated within the embedded two-character constituent and processed as a larger sublexical unit. The oculomotor system may therefore target an optimal viewing position near the center of this unit, rather than selecting each constituent character as a separate fixation target. By contrast, the first-level character combines with the embedded two-character constituent at a higher structural level and may be less tightly integrated with either of its individual characters. It may therefore function as a relatively independent and informative saccade target. Consequently, the second-level initial character in an LB and the second-level final character in an RB would be more likely to be skipped than the corresponding first-level characters.

The initial-character duration results provide qualified support for this account. Low-familiarity RBs showed reliably longer initial-character TFD than low-familiarity LBs. This later measure is compatible with greater processing demands for the initial character occupying the first-level role in RBs than for the initial character occupying the second-level role in LBs. Although cross-linguistic evidence suggests that an embedded bimorphemic constituent can function as a larger processing unit during trimorphemic word recognition (Bertram et al., 2011; Y. Song et al., 2019), the present TFD pattern is compatible with, rather than direct evidence for, such grouping.

The dissociation between final-character SP and fixation durations must be interpreted cautiously. One possible explanation is that structural grouping influenced parafoveal processing and saccade-target selection before the final character was directly fixated. Yan et al. (2026) showed that parafoveal grouping information can affect fixation placement and subsequent target processing during Chinese reading. Accordingly, some information about the final character and its structural relation to the preceding characters may have been obtained parafoveally, contributing to the branching difference in SP without necessarily producing a corresponding difference in fixation duration once the final character was directly fixated. High skipping may have further reduced the sensitivity of the duration analyses: final-character FFD, GD, and TFD were calculated only on directly fixated trials and were based on 1463–1465 observations, substantially fewer than the corresponding SP analysis. These analyses therefore had lower precision and were based on a selected subset of trials.

### 4.2. Familiarity and Predictability as Modulators

Initial-character fixation durations varied jointly with familiarity and branching, but the reliable follow-up pattern was confined to TFD: among low-familiarity H3CWs, initial-character TFD was longer in RBs than in LBs, and within RBs it was longer for low- than for high-familiarity items. The corresponding low-familiarity branching contrasts in FFD and GD were not significant after Holm adjustment. This pattern indicates that familiarity-dependent branching differences were most clearly expressed in later attention allocation to the initial character. One possible interpretation is that the contribution of structural information becomes more detectable when whole-word familiarity is low. Dual-route accounts provide a theoretical basis for this interpretation: weaker whole-word representations may increase the contribution of constituent information, whereas stronger representations may facilitate whole-word access (Baayen et al., 1997; Schreuder & Baayen, 1995). Accordingly, the hierarchical role of the initial character may be more consequential for attention allocation when the H3CW is less familiar, whereas greater familiarity may reduce the observable branching difference. However, the observed eye-movement differences are compatible with, but do not directly demonstrate, familiarity-dependent changes in the relative contributions of whole-word and constituent information.

Final-character processing also underscores the interpretive problem created by the stimulus imbalance. Predictability differed substantially between the familiarity conditions, and the apparent familiarity effects on final-character SP and TFD in the unadjusted sensitivity models were no longer significant in the primary models controlling for predictability. The data therefore do not provide evidence for familiarity effects on these measures that are independent of predictability. In the primary SP model, higher predictability increased final-character skipping in LBs, whereas no reliable predictability effect was detected in RBs; in TFD, higher predictability was associated with shorter durations. These findings indicate that predictability contributed to final-character processing, but statistical adjustment cannot correct the underlying imbalance between familiarity and predictability in the stimulus sets or fully disentangle their effects. The significant LB slope is compatible with the use of information from the preceding two-character constituent when readers decide whether to fixate the structurally independent final character. The weaker RB slope, where the final character is embedded within the two-character constituent, is also compatible with the grouping account proposed above: this character may be less likely to function as an independent saccade target and may therefore be less sensitive to its individual predictability. However, the present analysis did not directly measure constituent grouping or the information used during saccade-target selection. This possible account is represented by the predictability pathway in Figure 4.

Taken together, the findings indicate that character-level eye movements vary with branching structure, familiarity, and final-character predictability, although the evidence differs across measures. The opposite SP effects across positions are compatible with early sensitivity to the characters’ structural roles, whereas the reliable familiarity-dependent branching contrast was confined to initial-character TFD. From a resource-rational perspective, these patterns may reflect flexible attention allocation in response to structural and lexical information and processing demands (Butko & Movellan, 2008; Lieder & Griffiths, 2020). Because expected information gain and processing cost were not directly examined, this perspective is best regarded as a theoretical framework that organizes the present findings and generates testable predictions for future research.

## 5. Limitations

Several limitations constrain the interpretation of the findings. First, in binary-branching three-character words, hierarchical role is definitionally linked to branching direction and character position: the initial character is first-level in RBs and second-level in LBs, whereas the final character has the reverse mapping. These factors therefore cannot be fully separated within the present class of materials. Although the reversal of SP effects across positions is consistent with sensitivity to hierarchical role and reduces the plausibility of a position-invariant RB or LB advantage, explanations based on a Branching × Position relation or other position-specific oculomotor factors cannot be excluded. Future studies could adopt designs that vary structural interpretations while holding character identity and position constant, thereby providing a more direct test of hierarchical-role sensitivity.

Second, final-character predictability differed substantially between the familiarity conditions. Closer matching was constrained by the limited number of naturally occurring Chinese three-character words that met the structural and lexical selection criteria, making familiarity and predictability difficult to vary independently within the available item pool. Including predictability in the primary models adjusted for the measured covariance but could not eliminate the underlying stimulus imbalance. Consequently, the respective contributions of familiarity and predictability to final-character processing cannot be fully disentangled. Future studies could use larger norming pools to select more closely matched materials or, in a separate design, manipulate contextual support independently of lexical familiarity. Third, the proposed distinction between holistic and constituent-based processing was inferred indirectly from familiarity-related eye-movement differences. Because neither holistic lexical access nor constituent decomposition was directly measured, these processes should be regarded as possible explanations rather than demonstrated mechanisms. Similarly, the resource-rational account was not tested directly because expected information gain and processing cost were neither manipulated nor measured. Future research could operationalize these quantities to derive and test predictions that distinguish this framework from other accounts of lexical and oculomotor processing.

Fourth, final-character FFD, GD, and TFD were calculated only for directly fixated trials and were based on 1463–1465 observations, substantially fewer than the corresponding SP analysis. The high final-character skipping rate therefore reduced statistical precision and restricted the duration analyses to a selected subset of trials. The null duration effects should consequently be interpreted as an absence of reliable branching-related differences among directly fixated trials, rather than as evidence for or against hierarchical processing. Finally, the relatively large font used to define character-level interest areas may have prevented the entire H3CW, particularly its final character, from falling fully within parafoveal vision. Future studies of parafoveal grouping could use smaller characters and designs that yield more directly fixated final-character trials. An additional limitation is that the participants were young university students, which may limit the generalizability of the findings to readers of other ages, educational backgrounds, or levels of reading experience.

## 6. Conclusions

In summary, character-level eye movements in Chinese H3CWs varied with branching structure, familiarity, and final-character predictability. The reversal of the branching effect in initial- and final-character skipping is compatible with early sensitivity to the structural roles associated with branching, whereas the fixation-duration evidence was more limited and was confined mainly to the familiarity-dependent pattern in initial-character TFD. Predictability contributed to final-character processing, whereas familiarity effects independent of predictability were not supported. The findings support a tentative structure-sensitive interpretation of attention allocation and provide a basis for future tests of the mechanisms underlying H3CW recognition.

## Figures and Tables

**Table 1:** Critical materials’ feature scores.

	High-Familiarity RB		High-Familiarity LB		Low-Familiarity RB		Low-Familiarity LB	
	*M*	*SD*	*M*	*SD*	*M*	*SD*	*M*	*SD*
H3CW Familiarity	5.97	0.46	6.06	0.46	3.82	0.83	3.99	0.56
H3CW Non-literalness	2.29	0.79	2.25	0.73	2.36	0.72	2.47	0.67
Predictability	0.66	0.27	0.68	0.18	0.32	0.27	0.26	0.22
WC Frequency	2.18	0.58	2.25	0.31	2.19	0.48	2.20	0.71
WC Familiarity	5.24	0.70	5.21	0.55	5.11	0.66	5.00	0.59
WC Non-literalness	2.42	0.57	2.56	0.49	2.44	0.55	2.60	0.53
C1 Frequency	3.98	0.55	3.80	0.74	3.97	0.61	3.89	0.68
C1 IPP	0.63	0.20	0.62	0.18	0.54	0.23	0.56	0.18
C1 NoS	8.00	3.06	8.32	2.58	8.74	3.11	7.83	2.95
C2 Frequency	4.24	0.65	4.06	0.47	4.19	0.56	4.03	0.52
C2 IPP	0.43	0.19	0.41	0.12	0.49	0.19	0.41	0.18
C2 FPP	0.42	0.16	0.43	0.11	0.36	0.14	0.43	0.15
C2 NoS	7.92	2.54	7.75	2.72	7.09	2.35	7.70	3.02
C3 Frequency	3.87	0.71	4.15	0.70	4.05	0.49	3.97	0.73
C3 FPP	0.63	0.20	0.57	0.14	0.54	0.21	0.59	0.21
C3 NoS	8.33	2.75	8.75	2.98	8.17	2.29	8.83	2.37

*Note*. WC = the two-character word constituent, C1 = the initial character, C2 = the middle character, C3 = the final character, IPP = the initial positional probability, FPP = the final positional probability, NoS = number of strokes.

**Table 2:** Eye movement measures for the initial and final characters.

Character Position	Familiarity	Branching	SP	*n*	FFD	*n*	GD	*n*	TFD	*n*
Initial	High	RB	0.50 (0.01)	1833	210 (3)	904	218 (4)	897	246 (4)	1148
	High	LB	0.54 (0.01)	2181	209 (3)	990	218 (3)	986	238 (3)	1273
	Low	RB	0.52 (0.01)	1490	213 (4)	705	227 (5)	702	305 (6)	1035
	Low	LB	0.54 (0.01)	1637	204 (4)	739	212 (4)	739	257 (5)	942
Final	High	RB	0.85 (0.01)	1833	237 (6)	261	244 (7)	261	259 (10)	260
	High	LB	0.81 (0.01)	2181	246 (6)	391	254 (6)	391	263 (7)	393
	Low	RB	0.81 (0.01)	1490	242 (7)	264	257 (8)	264	284 (10)	264
	Low	LB	0.66 (0.01)	1637	244 (5)	547	267 (5)	547	292 (6)	548

*Note*. Standard errors are provided in parentheses. The reported *n* values are the numbers of observations included in the final models after all trial- and measure-level exclusions.

**Table 3:** Mixed-effects analyses for the initial and final characters in the primary models.

	Effect	SP				FFD				GD				TFD			
		*b*	*SE*	*z*	*p*	*b*	*SE*	*t*	*p*	*b*	*SE*	*t*	*p*	*b*	*SE*	*t*	*p*
Initial	(Intercept)	0.16	0.12	1.33	0.184	5.28	0.03	**183.89**	**<0.001**	5.31	0.03	**168.40**	**<0.001**	5.42	0.04	**140.50**	**<0.001**
	Fml	0.05	0.07	0.71	0.479	0.01	0.02	0.40	0.691	0.01	0.02	0.34	0.738	0.12	0.03	**3.59**	**<0.001**
	Brn	−0.13	0.05	**−2.49**	**0.013**	0.01	0.01	0.74	0.464	0.01	0.02	0.79	0.434	0.09	0.03	**3.24**	**0.002**
	Fml × Brn	0.10	0.11	0.98	0.329	0.05	0.03	**2.10**	**0.038**	0.07	0.03	**2.13**	**0.036**	0.14	0.05	**2.76**	**0.007**
	Contrast 1	−	−	−	−	−0.04	0.02	−1.91	0.118	−0.04	0.02	−1.97	0.103	−0.16	0.04	**−4.10**	**<0.001**
	Contrast 2	−	−	−	−	0.03	0.02	1.63	0.118	0.04	0.02	1.60	0.113	0.19	0.04	**4.59**	**<0.001**
	Pred	0.01	0.11	0.09	0.930	0.02	0.03	0.89	0.378	0.02	0.03	0.48	0.629	0.03	0.06	0.49	0.628
Final	(Intercept)	1.38	0.27	**5.20**	**<0.001**	5.39	0.03	**159.55**	**<0.001**	5.44	0.04	**146.90**	**<0.001**	5.49	0.04	**123.83**	**<0.001**
	Fml	−0.30	0.26	−1.20	0.232	−0.02	0.03	−0.49	0.625	0.01	0.03	0.20	0.840	0.12	0.07	1.58	0.118
	Brn	1.47	0.36	**4.03**	**<0.001**	−0.02	0.02	−1.00	0.320	−0.04	0.03	−1.44	0.152	−0.03	0.07	−0.43	0.672
	Fml × Brn	−	−	−	−	−0.02	0.05	−0.40	0.694	−0.04	0.05	−0.71	0.478	0.02	0.15	0.13	0.894
	Pred	1.19	0.41	**2.92**	**0.004**	−0.04	0.05	−0.89	0.378	−0.09	0.06	−1.66	0.101	−0.15	0.06	**−2.25**	**0.027**
	Brn × Pred	−1.58	0.64	**−2.48**	**0.013**	−	−	−	−	−	−	−	−	−0.03	0.13	−0.21	0.832
	Fml × Pred	−	−	−	−	−	−	−	−	−	−	−	−	−0.17	0.13	−1.34	0.183
	Fml × Brn × Pred	−	−	−	−	−	−	−	−	−	−	−	−	−0.13	0.26	−0.49	0.629

*Note*. Significant terms are shown in bold. SP was analyzed using logistic generalized linear mixed-effects models; FFD, GD, and TFD were analyzed using linear mixed-effects models. *b* = regression coefficient. Contrast 1 tests the branching effect among low-familiarity H3CWs, and Contrast 2 tests the familiarity effect among RBs. The *p* values for the planned contrasts are Holm-adjusted; all other *p* values are unadjusted omnibus-test values. Fml = familiarity; Brn = branching; Pred = predictability.

## Data Availability

The datasets generated and analyzed during the current study are available at https://osf.io/6ep2q/ (accessed on 5 September 2026).

## Appendix A

**Table A1:** Trial- and measure-level exclusions by experimental condition. (**a**) Trial-level exclusions. (**b**) Measure-specific z-score filtering.

(**a**)						
**Condition**	**Trials Before Exclusion, *n***	**Incorrect Responses,** ***n* (%)**	**Blinks,** ***n* (%)**	**RT < 300 ms,** ***n* (%)**	**Total Excluded,** ***n* (%)**	**Retained Trials,** ***n* (%)**
High-familiarity RB	2184	33 (1.51%)	317 (14.51%)	1 (0.05%)	351 (16.07%)	1833 (83.93%)
High-familiarity LB	2548	24 (0.94%)	343 (13.46%)	0 (0.00%)	367 (14.40%)	2181 (85.60%)
Low-familiarity RB	2093	280 (13.38%)	323 (15.43%)	0 (0.00%)	603 (28.81%)	1490 (71.19%)
Low-familiarity LB	2093	152 (7.26%)	302 (14.43%)	2 (0.10%)	456 (21.79%)	1637 (78.21%)
Overall	8918	489 (5.48%)	1285 (14.41%)	3 (0.03%)	1777 (19.93%)	7141 (80.07%)
(**b**)						
**Condition**	**Initial FFD,** ***n* (%)**	**Initial GD,** ***n* (%)**	**Initial TFD,** ***n* (%)**	**Final FFD,** ***n* (%)**	**Final GD,** ***n* (%)**	**Final TFD,** ***n* (%)**
High-familiarity RB	8 (0.88%)	15 (1.64%)	19 (1.63%)	13 (4.74%)	13 (4.74%)	14 (5.11%)
High-familiarity LB	12 (1.20%)	16 (1.60%)	12 (0.93%)	16 (3.93%)	16 (3.93%)	14 (3.44%)
Low-familiarity RB	7 (0.98%)	10 (1.40%)	4 (0.38%)	15 (5.38%)	15 (5.38%)	15 (5.38%)
Low-familiarity LB	9 (1.20%)	9 (1.20%)	14 (1.46%)	11 (1.97%)	11 (1.97%)	10 (1.79%)
Overall	36 (1.07%)	50 (1.48%)	49 (1.10%)	55 (3.62%)	55 (3.62%)	53 (3.49%)

*Note*. (a) reports sequential, mutually exclusive trial-level exclusions. (b) reports measure-specific exclusions of observations with |z| ≥ 3 or undefined within-participant, within-condition z scores; percentages are based on eligible directly fixated observations before this filtering. Final model sample sizes are reported in Table 2.

**Table A2:** Final fixed- and random-effects structures for the primary and sensitivity models.

Character Position	Analysis	Model Component	Measure	Final Fixed- and Random-Effects Structure
Initial	Primary	Omnibus	SP	Fml × Brn + pred + (1|subject)
			FFD	Fml × Brn + pred + (1|subject) + (1|item)
			GD	Fml × Brn + pred + (1|subject) + (1|item)
			TFD	Fml × Brn + pred + (1 + Fml + Brn|subject) + (1|item)
		Planned contrasts	FFD	condition + pred + (1|subject) + (1|item)
			GD	condition + pred + (1|subject) + (1|item)
			TFD	condition + pred + (1 + Fml + Brn|subject) + (1|item)
	Sensitivity	Omnibus	SP	Fml × Brn + (1|subject)
			FFD	Fml × Brn + (0 + Fml|subject) + (1|item)
			GD	Fml × Brn + (1|subject) + (1|item)
			TFD	Fml × Brn + (0 + Fml × Brn|subject) + (1|item)
		Planned contrasts	FFD	condition + (0 + Fml|subject) + (1|item)
			GD	condition + (1|subject) + (1|item)
			TFD	condition + (1 + Fml + Brn|subject) + (1|item)
Final	Primary	Omnibus	SP	Fml + Brn × pred + (1 + Fml|subject) + (1|item)
			FFD	Fml × Brn + pred + (1|subject) + (1|item)
			GD	Fml × Brn + pred + (1|subject) + (1|item)
			TFD	Fml × Brn × pred + (1 + Fml|subject) + (1|item)
		Simple slopes	SP	Estimated directly from the primary omnibus GLMM using emtrends(); no separate model was fitted
	Sensitivity	Omnibus	SP	Fml × Brn + (1 + Fml|subject) + (1|item)
			FFD	Fml × Brn + (1|subject) + (1|item)
			GD	Fml × Brn + (1|subject) + (1|item)
			TFD	Fml × Brn + (1 + Fml|subject) + (1|item)

*Note*. Models with and without predictability were treated as primary and sensitivity analyses, respectively. Initial-character planned contrasts were obtained from models using the four-level condition factor; final-character SP slopes were estimated using emtrends() function in the emmeans package. Fml = familiarity; Brn = branching; pred = predictability.

**Table A3:** Mixed-effects analyses for the initial and final characters in the sensitivity models excluding predictability.

	Effect	SP				FFD				GD				TFD			
		*b*	*SE*	*z*	*p*	*b*	*SE*	*t*	*p*	*b*	*SE*	*t*	*p*	*b*	*SE*	*t*	*p*
Initial	(Intercept)	0.17	0.11	1.53	0.126	5.29	0.03	**206.27**	**<0.001**	5.32	0.03	**195.72**	**<0.001**	5.43	0.03	**196.20**	**<0.001**
	Familiarity	0.04	0.05	0.83	0.407	0.00	0.01	−0.05	0.965	0.00	0.02	0.05	0.962	0.11	0.03	**4.15**	**<0.001**
	Branching	−0.13	0.05	**−2.49**	**0.013**	0.01	0.01	0.80	0.424	0.01	0.02	0.81	0.421	0.09	0.03	**3.24**	**0.002**
	Fml × Brn	0.10	0.10	0.99	0.323	0.05	0.03	**2.17**	**0.032**	0.07	0.03	**2.19**	**0.031**	0.15	0.05	**2.80**	**0.006**
	Contrast 1	−	−	−	−	−0.04	0.02	−2.00	0.096	−0.05	0.02	−2.02	0.092	−0.16	0.04	**−4.17**	**<0.001**
	Contrast 2	−	−	−	−	0.03	0.02	1.47	0.144	0.03	0.02	1.55	0.124	0.19	0.04	**4.90**	**<0.001**
Final	(Intercept)	1.95	0.18	**10.68**	**<0.001**	5.37	0.02	**216.89**	**<0.001**	5.40	0.03	**202.24**	**<0.001**	5.44	0.03	**188.47**	**<0.001**
	Familiarity	−0.78	0.21	**−3.75**	**<0.001**	0.00	0.02	0.05	0.960	0.04	0.03	1.50	0.136	0.09	0.03	**2.84**	**0.006**
	Branching	0.73	0.20	**3.71**	**<0.001**	−0.03	0.02	−1.07	0.289	−0.04	0.03	−1.53	0.129	−0.04	0.03	−1.35	0.181
	Fml × Brn	0.72	0.40	1.83	0.068	−0.02	0.05	−0.50	0.619	−0.05	0.05	−0.90	0.370	−0.05	0.06	−0.80	0.428

*Note*. Significant terms are shown in bold. SP was analyzed using logistic generalized linear mixed-effects models; FFD, GD, and TFD were analyzed using linear mixed-effects models. Contrast 1 tests the branching effect among low-familiarity H3CWs, and Contrast 2 tests the familiarity effect among RBs. The *p* values for the planned contrasts are Holm-adjusted; all other *p* values are unadjusted omnibus-test values.
